# Combining Engineering Precision with Clinical Relevance: A Novel Dual Framework for Assessing Pedicle Screw Accuracy in Spine Surgery

**DOI:** 10.3390/jcm15062328

**Published:** 2026-03-18

**Authors:** Arnaud Delafontaine, Olivier Cartiaux, Bernard G. Francq, Virginie Cordemans

**Affiliations:** 1 CIAMS, Université Paris-Saclay, 91404 Orsay, France; 2 Laboratoire D’Anatomie Fonctionnelle, Faculté des Sciences de la Motricité, Université Libre de Bruxelles, 1070 Bruxelles, Belgium; 3 Haute Ecole ICHEC-ECAM-ISFSC, ECAM Brussels Engineering School, Promenade de l’Alma 50, 1200 Brussels, Belgium; olivier.cartiaux@gmail.com; 4 Institut de Statistique, Biostatistique et Sciences Actuarielles (ISBA), Université Catholique de Louvain (UCL), Voie du Roman Pays 20, 1348 Louvain-la-Neuve, Belgium; bernard.x.francq@gsk.com; 5 Orthopedic Surgery and Traumatologic Department, Hospital University Center Brugmann, Place A. Van Gehuchten, 1020 Brussels, Belgium; virginie.cordemans@chu-brugmann.be

**Keywords:** pedicle screw accuracy, spine surgery, coaxiality, pedicle wall distance, cone beam computed tomography, surgical navigation

## Abstract

**Background/Objectives:** Accurate pedicle screw placement is critical in spine surgery, as malposition can cause neurological, vascular, or visceral injuries and compromise construct stability. The primary objective of this study was to develop and experimentally validate a dual quantitative framework for assessing pedicle screw placement accuracy, combining (1) coaxiality, a standardized geometric metric of trajectory alignment, and (2) pedicle wall distance (dpw), a novel parameter defined as the minimal distance between the screw axis and the pedicle cortex providing surgeons with direct, millimetric, clinically actionable feedback. A secondary objective was to compare these parameters: dpw, coaxiality, entry point errors and orientation angle errors between senior surgeons and residents to evaluate the influence of surgical experience. We hypothesized that this framework would provide reproducible quantitative measurements, demonstrate strong agreement with established CBCT-based grading systems, and allow meaningful subgroup comparisons by experience level. **Methods:** Eight operators (four senior surgeons, four residents) performed 240 pedicle screw insertions on synthetic polyurethane lumbar spine models using freehand, CBCT-assisted, and navigation-assisted techniques. Predefined 3D trajectories were compared with actual screw positions digitized with sub-millimetric precision. Errors, coaxiality, and dpw were computed, and dpw was validated against CBCT-based Gertzbein and Heary classifications. Agreement and diagnostic performance metrics (Kappa, sensitivity, specificity) were calculated. **Results:** Of 236 analyzable screws, coaxiality correlated with entry point errors (ρ = 0.41), target point errors (ρ = 0.85), and orientation angle errors (ρ = 0.48), confirming its robustness as an engineering metric. dpw provided immediate, interpretable feedback and demonstrated near-perfect agreement with CBCT grading (Kappa = 0.86; sensitivity = 0.96; specificity = 0.97), detecting breaches missed by qualitative classifications. Subgroup analyses indicated small but significant differences between senior and junior surgeons for target point errors (*p* = 0.006), orientation angle errors (*p* = 0.025), and coaxiality (*p* = 0.023), whereas entry point errors (*p* = 0.201) and dpw (*p* = 0.163) did not differ significantly. **Conclusions:** This dual-metric framework bridges engineering rigor and intraoperative applicability. Coaxiality supports reproducible research assessment, while dpw enables actionable surgical feedback. The framework allows objective comparison across operators of different experience levels. Together, these metrics offer a standardized, clinically relevant, and quantitative method for evaluating pedicle screw placement, with potential to enhance surgical safety, education, and patient outcomes.

## 1. Introduction

In spine surgery, accurate pedicle screw insertion is critical, as malposition can lead to spinal cord, visceral, or vascular injuries, compromising both patient safety and construct stability [1,2,3,4]. Ideally, screws should be fully contained within the pedicle, as described in several studies, to minimize complications and ensure optimal fixation [1,2,5].

Since the 1990s, various assistive technologies have been developed to improve screw placement accuracy, including two-dimensional (2D) and three-dimensional (3D) fluoroscopy, computed tomography (CT), interventional magnetic resonance imaging (MRI), navigation systems, robotic devices, 3D-printed patient-specific guides, and hybrid approaches. Numerous studies have demonstrated the benefits of these technologies in achieving safer and more precise screw placement [6,7,8,9].

Several screw insertion techniques are currently used in clinical practice, each with specific advantages and limitations documented in the literature. Traditional freehand placement, relying on anatomical landmarks, remains widely practiced due to its low cost and minimal infrastructure requirements. However, reported accuracy rates vary widely, often between 70 and 95% depending on surgeon experience and spinal level, with higher misplacement rates particularly in thoracic segments [10].

Fluoroscopy-guided techniques improve visualization compared with freehand alone and achieve moderately high accuracy, but still expose patients and operating room staff to radiation and offer only indirect two-dimensional guidance. Systematic reviews have shown fluoroscopy accuracy rates typically in the range of ~80–92%, lower than navigation methods [11].

Navigation-assisted methods (CT or 3D fluoroscopy-based intraoperative guidance) consistently demonstrate higher placement accuracy (often >95% clinically acceptable screws), with meta-analyses reporting improved precision compared with fluoroscopy or freehand techniques [12]. These systems also tend to reduce intraoperative radiation exposure to surgical teams and improve screw placement reliability across complex anatomy [11].

Robotic-assisted techniques further refine image-guided approaches by providing stable, preplanned trajectories and mechanical guidance. Multiple meta-analyses have found robotic and navigation-assisted placement to be significantly associated with higher odds of accurate screw placement (e.g., OR ~2.6–2.8 for achieving acceptable screw position versus fluoroscopic freehand) and lower rates of major complications, facet joint violations, and revisions [13]. Some recent Bayesian network meta-analyses [14] also suggest that robotic assistance may offer the highest accuracy among freehand, navigation, and robotic methods, albeit sometimes with longer operative times.

Patient-specific 3D printed drill guides have been shown to improve pedicle screw placement accuracy, with clinical series reporting very high rates of screws within the safe zone and minimal cortical breach when compared to preoperative plans based on CT imaging. For example, Cool et al. [15] demonstrated that all pedicle screws placed using such 3D-printed guides were accurately positioned without relevant breaches on postoperative CT analysis.

Augmented reality (AR) navigation systems in spine surgery have similarly shown promising accuracy. Clinical and cadaveric studies report pedicle screw placement accuracy in the range of approximately 90–95% for AR-assisted methods, with some systems achieving comparable results to advanced navigation or robotic assistance [16].

Despite these trends, direct comparisons remain challenging due to heterogeneity in outcome definitions, imaging evaluation protocols, learning curves, and clinical endpoints, reinforcing the need for standardized, quantitative evaluation frameworks.

Cordemans et al. [17] focused on evaluating a novel intraoperative 3D imaging system for pedicle screw guidance, assessment, or integration with navigation systems. This system, an intraoperative cone beam CT (CBCT) combined with an interventional angiographic robot (Artis zeego II, Siemens Healthcare, Forchheim, Germany), has been adapted for spine surgery [18,19].

Clinical studies have shown its feasibility in assisting screw placement [17] and intraoperative assessment, with the advantage of allowing immediate repositioning of misplaced screws and reducing reoperation rates [20].

Despite these advances, defining surgical accuracy and comparing results remains challenging due to heterogeneous evaluation methods. Most current approaches rely on qualitative classifications, such as Gertzbein–Robbins [21] and Heary [22], which are subject to interobserver variability.

The Gertzbein–Robbins scale, initially described in 1990 [21], grades pedicle screw accuracy according to the extent of cortical breach visible on computed tomography. They were the first authors to propose a classification of pedicle breaches, in particular breach in the medial part of the pedicles graded with increments of 2 mm according to four grades from 0 to 3.

The Heary classification [22], introduced in 2004 for thoracic pedicle screw assessment, categorizes screw placement based on the direction and clinical relevance of cortical wall violation. It differentiates screws fully contained within the pedicle from those breaching the cortex medially, laterally, superiorly, inferiorly, or anteriorly, and considers the potential neurological or visceral risk associated with each breach pattern.

Although widely adopted, both systems remain semi-quantitative and rely on subjective radiological interpretation, which may introduce interobserver and intraobserver variability. Moreover, they focus primarily on cortical breach rather than providing a continuous three-dimensional deviation from a preoperative plan, thereby limiting fine-grained comparison between surgical techniques.

Some authors have proposed quantitative assessments using 2D/3D geometric parameters (linear and angular) to measure deviations from planned screw trajectories [23,24,25]. However, these methods are often ad hoc, locally applied, and difficult to compare across studies.

The most recent study by Byeong-Jin et al. [26] employed a 3D quantitative method to calculate offsets between planned and inserted screw trajectories, assessing target, entry point, depth, and angulation. Their methodology involved segmentation of pre- and postoperative vertebrae and paired-point registration for alignment. Limitations included the lack of validation against established clinical classifications, such as Gertzbein–Robbins [21], and ambiguity regarding manual versus automatic segmentation, manual offering higher precision but operator dependence, and automatic improving reproducibility at potential cost of accuracy.

Therefore, despite major advances in surgical guidance technologies, there is currently no standardized, validated, and reproducible quantitative framework allowing objective cross-platform comparison of pedicle screw placement accuracy.

The primary objective of this study was to develop and validate a novel quantitative evaluation framework for pedicle screw placement accuracy based on ISO-standardized geometric parameters and pedicle wall distance measurements. This framework was designed to enable continuous three-dimensional deviation analysis and facilitate objective comparison with established qualitative grading systems.

Emerging technologies such as AI-driven segmentation and automated trajectory planning increasingly rely on precise quantitative metrics like coaxiality and dpw to guide and evaluate screw placement, underlining the relevance of such frameworks for both research and clinical applications.

We hypothesized that the proposed quantitative method would demonstrate strong correlation with established clinical classifications (Gertzbein–Robbins [21] and Heary [22]) while providing improved precision, reproducibility, and discriminatory capacity compared with traditional semi-quantitative grading systems.

A secondary objective was to compare these parameters: dpw, coaxiality, entry point errors (i.e., minimal distance from the actual screw entry point to the desired axis), and orientation angle errors (i.e., angular difference between the actual screw vector and the desired axis) between senior surgeons and residents to evaluate the influence of surgical experience.

## 2. Material and Methods

### 2.1. Sample and Geometrical Model

**Figure 1:**
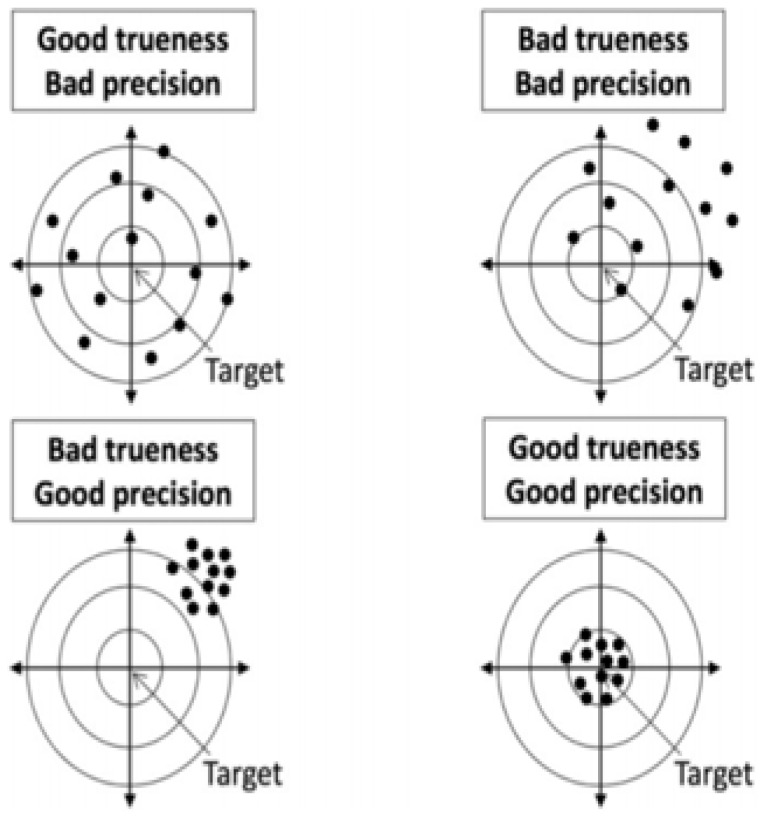
Trueness and Precision (ISO 5725-1).

In the literature, the terms “precision” and “accuracy” are often used interchangeably. To standardize terminology, we adopted ISO 5725-1 (International Standardization Organization) [27], which defines accuracy as a combination of a systematic error or bias component (trueness) and a random error component (precision) (Figure 1).

Trueness is defined as the closeness between the mean value obtained from a large series of surgical maneuvers and the intended surgical target. Precision reflects the closeness among independent surgical gestures performed under the same conditions. Therefore, accuracy corresponds to the closeness of an achieved surgical gesture to the desired surgical target. These ISO parameters have been applied in orthopedic surgery to objectively define the expected performance of surgical actions.

**Figure 2:**
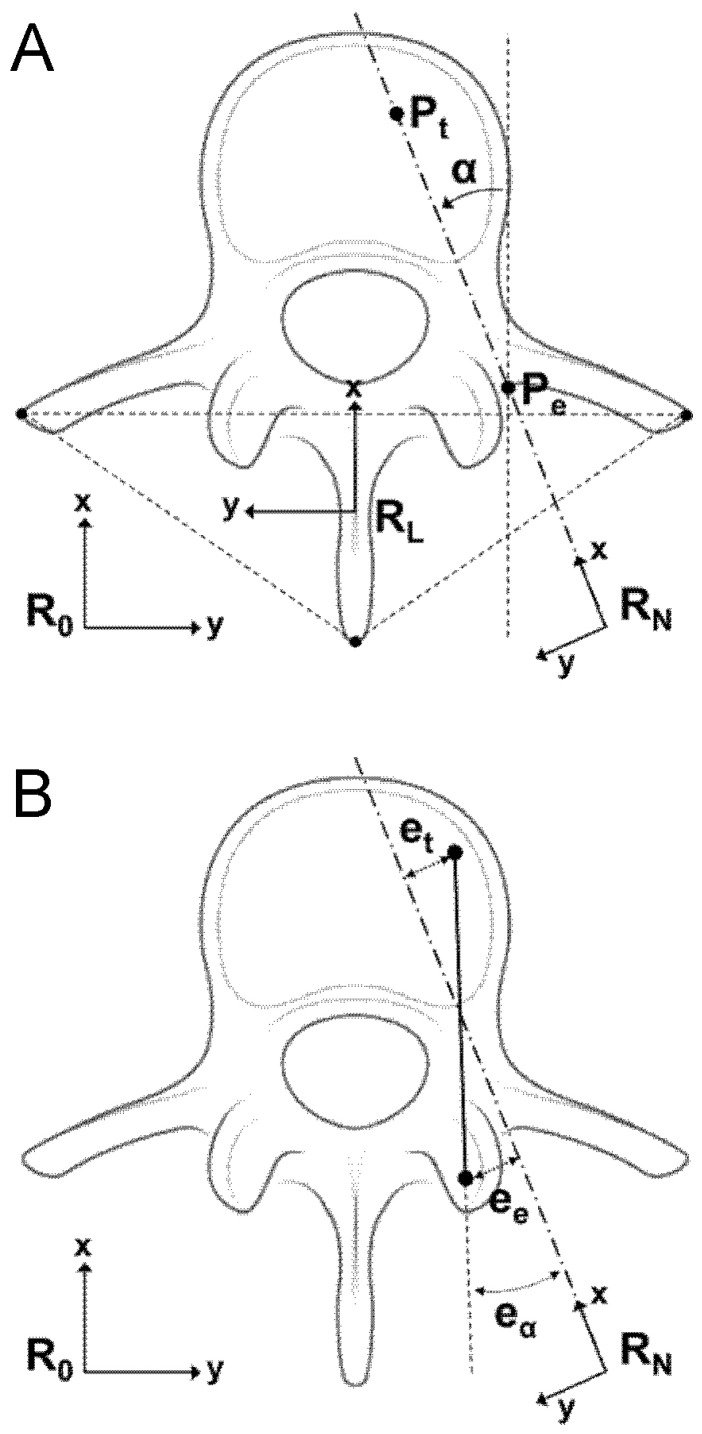
New geometrical model. (**A**) Definition of a desired positioning of a screw after insertion by a minimal set of two independent parameters: an entry point P_e_ and a target point P_t_, with the coordinates stated in the reference frame R_0_ of the CBCT images. The orientation α of the screw is defined by the direction vector from P_e_ to P_t_. A reference frame (R_L_) fixed to the vertebra is defined using three anatomical landmarks (spinous process and left and right transverse processes). A reference frame (R_N_) fixed to the screw is defined so as the x coordinate axis was aligned with the orientation of the screw. (**B**) A reference frame (R_N_) fixed to the screw is defined so as the x coordinate axis was aligned with the orientation of the screw. The error in entry point e_e_ is defined as the minimal distance (mm) from P_e_ to the x-axis of R_N_. The error in target point e_t_ is defined as the minimal distance (mm) from P_t_ to the x-axis of R_N_. The error in orientation angle e_α_ is defined as the angular difference in degrees between the direction vector from P_e_ to P_t_ and the x-axis of R_N_.

We propose a new geometrical model for pedicle screw positioning based on ISO 5725-1 (Figure 2). The desired screw position is defined using two independent parameters: Pe (entry point) and Pt (target point). Screw orientation is described by the vector from Pe to Pt, and the 3D orientation angle α (in degrees) is also calculated. Using this model, the following insertion errors between the actual screw and the desired trajectory can be measured. The Entry point error (ee, mm) defined as the minimal distance from the actual screw entry point to the desired axis (RN). The Target point error (et, mm) corresponded to a minimal distance from the actual screw tip to the desired axis (RN). The Orientation angle error (eα) represented by an angular difference between the actual screw vector and the desired axis (RN).

To simplify assessment and improve objectivity, we defined two additional parameters. The Coaxiality (C, mm) in line with ISO 1101-based measure of geometrical accuracy, defined as the maximum distance between the axis of the inserted screw and the desired trajectory [28].

**Figure 3:**
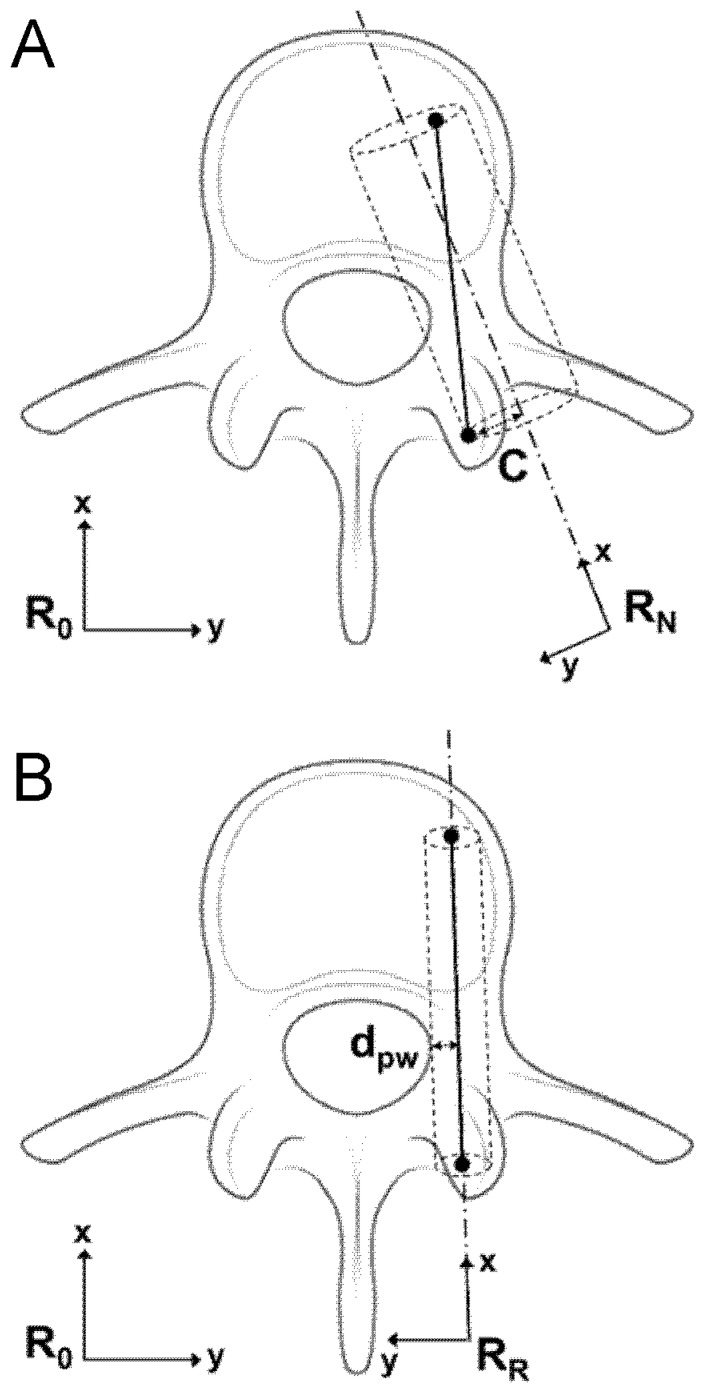
Representation of insertion errors, Coaxiality (**A**) and distance of the pedicle wall (**B**). (**A**) ISO-based coaxiality parameter C (mm) for the evaluation of screw insertion accuracy. The inserted screw is the solid line segment; the desired trajectory is the dashed line. The coaxiality is defined as the maximum distance (mm) between the axis of the inserted screw and the desired trajectory. (**B**) The distance of the pedicle wall (dpw) evaluates the position of the inserted screw relative to the boundary of the pedicle wall. The parameter dpw is defined as the minimal distance (mm) between the axis of the inserted screw and the boundary of the pedicle wall. (**B**) Knowing the diameter of the inserted screw, a dpw lower than radius of the screw represents a pedicle breach while a value of dpw greater than the radius of the screw represents absence of pedicle breach.

The Pedicle wall distance (d_pw_, mm) defined as the minimal distance between the screw axis and the pedicle wall. A d_pw_ smaller than the screw radius indicates a pedicle breach, while a dpw larger than the radius indicates no breach (Figure 3).

**Figure 4:**
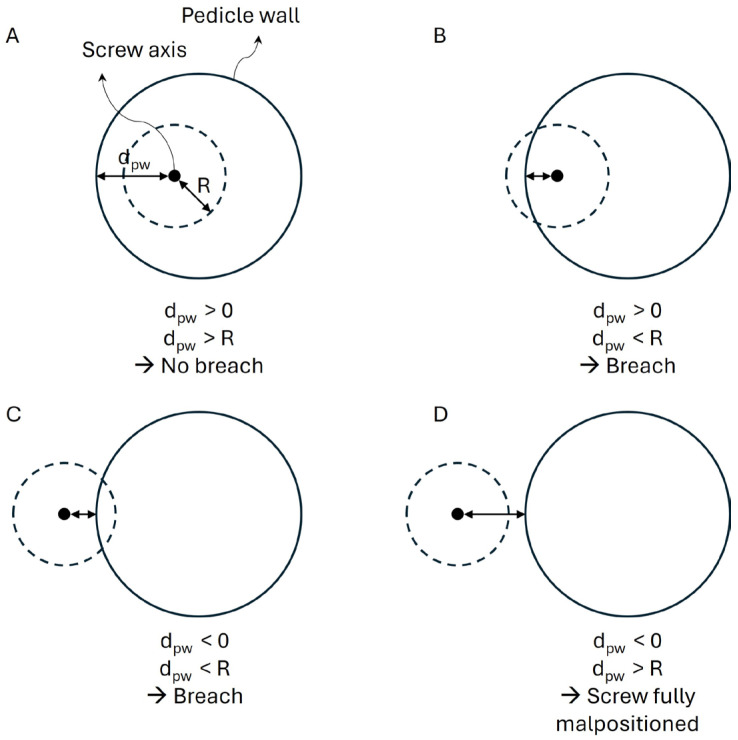
Schematization using an axial view of the screw inserted into the pedicle. The black dot represents the axis of the screw. The solid circle represents the pedicle wall. R is the radius of the screw. Distance to the pedicle wall is considered positive if the black dot is inside the black circle and negative if the black dot is outside the black circle. (**A**) dpw is positive and larger than the screw radius R, indicating a well-positioned screw with no breach. (**B**) d_pw_ is positive and smaller than R, indicating a breach. (**C**) d_pw_ is negative and smaller than R, also indicating a breach. (**D**) dpw is negative and larger than R, indicating a fully malpositioned screw.

To clarify the calculation of the d_pw_ parameter, particularly in cases where the screw is completely outside the pedicle (negative values), Figure 4 provides a schematic axial representation illustrating the geometric definition of dpw and its relationship to the screw radius (R), including the condition where d_pw_ < 0 and d_pw_ < R corresponding to a fully extra-pedicular screw without cortical contact.

### 2.2. Study Design

Eight operators, four experienced surgeons (senior) and four residents (junior), performed simulated pedicle screw insertions according conventional, CBCT assisted and navigation assisted insertion. Experiments were conducted using synthetic lumbar spine models (L1 to sacrum) made from solid rigid polyurethane closed foam [29]. The models included articulated anterior and posterior naugahyde ligaments and flexible disks, with a dimensional tolerance of 0.5 mm according to the manufacturer. The study was conducted exclusively in Belgium and is therefore a monocentric Belgian study, with no participating sites in France.

### 2.3. Intervention

**Figure 5:**
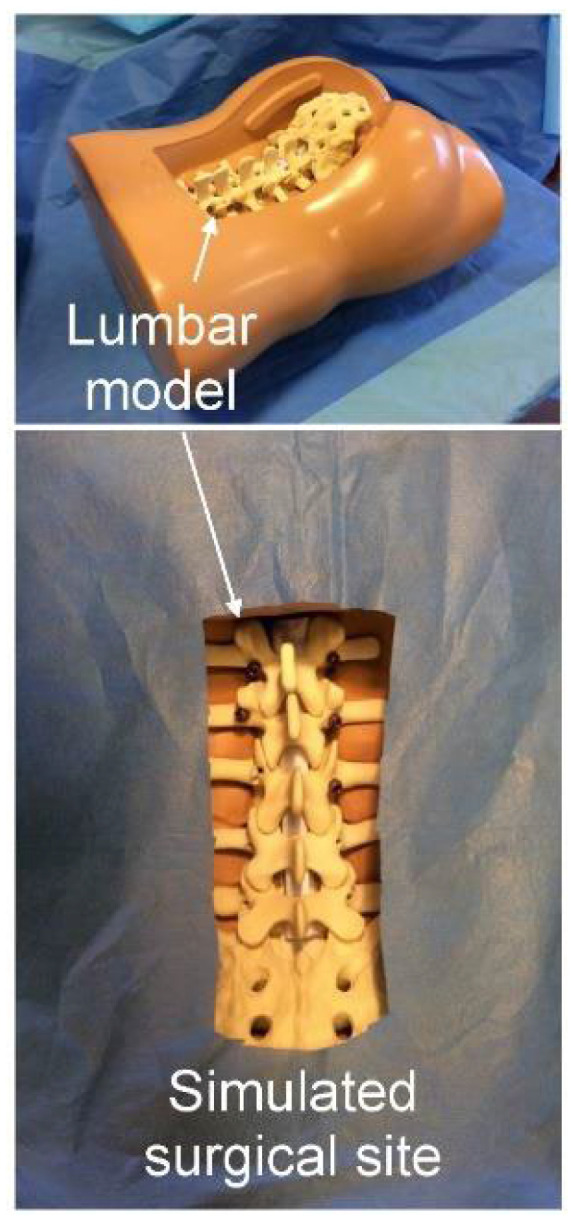
The lumbar spine model held in a soft tissues spine holder in prone position. The surgical site was covered with a blue drape to simulate realistic condition.

Screw insertions were performed using conventional surgical instruments (bone rongeurs, pedicle awls and probes, screwdrivers) and USS-II Polyaxial Pedicle Screws (45 mm length, 5 mm diameter; DePuy Synthes, Oberdorf, Switzerland). Models were positioned prone in a soft tissue spine holder, and the spinal canal was simulated using a roll of paper inside the vertebral foramen. The surgical field was draped with a blue cloth to mimic realistic operative conditions (Figure 5).

**Figure 6:**
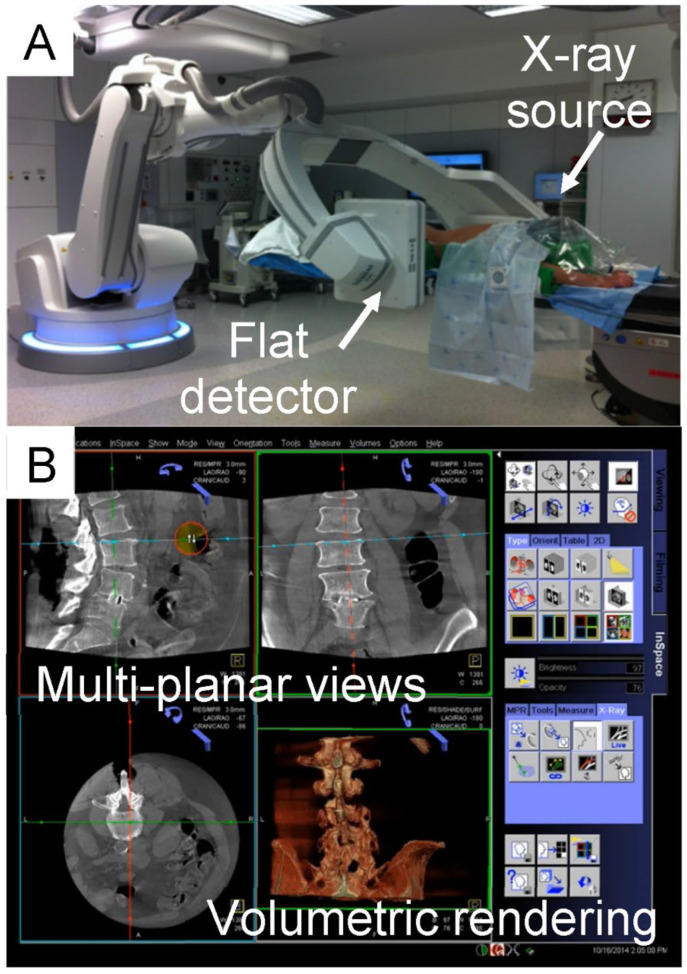
Illustration of the Artis zeego imaging system and simulated intraoperative use. (**A**) Intraoperative setup with the Artis zeego system positioned prior to image acquisition. The Artis zeego system is capable of moving around the patient, continuously acquiring fluoroscopic images. (**B**) Screen image available intraoperatively in the operating room, showing axial, sagittal, and coronal views as well as 3D view.

Each test bed was scanned using an intraoperative CBCT imaging system (Artis Zeego II, Siemens Healthcare, Forchheim, Germany), a floor-mounted multi-axis robotic C-arm (Figure 6A) equipped with syngo DynaCT software (version VB21). This system functions as both standard 2D fluoroscopy and a rapid 3D CBCT-like imaging modality. The large flat-panel detector (300 × 400 mm) acquires volumetric data by isocentric rotation around the model over 200°. The default 3D acquisition mode for spinal surgery captures 397 images during a six-second spin (6sDCT). During acquisition, the operator and staff leave the room to minimize X-ray exposure moving to a dedicated, lead-shielded room with visual monitoring of the patient.

Acquired images are automatically transferred to a dedicated workstation (syngo X Workplace, Siemens Healthcare, Forchheim, Germany) and reconstructed into volumetric data with isotropic 0.5 mm voxels using a filtered backprojection algorithm. Volumetric data can be visualized intraoperatively as multi-planar views or volumetric renderings (Figure 6B), allowing detailed assessment of screw placement.

**Figure 7:**
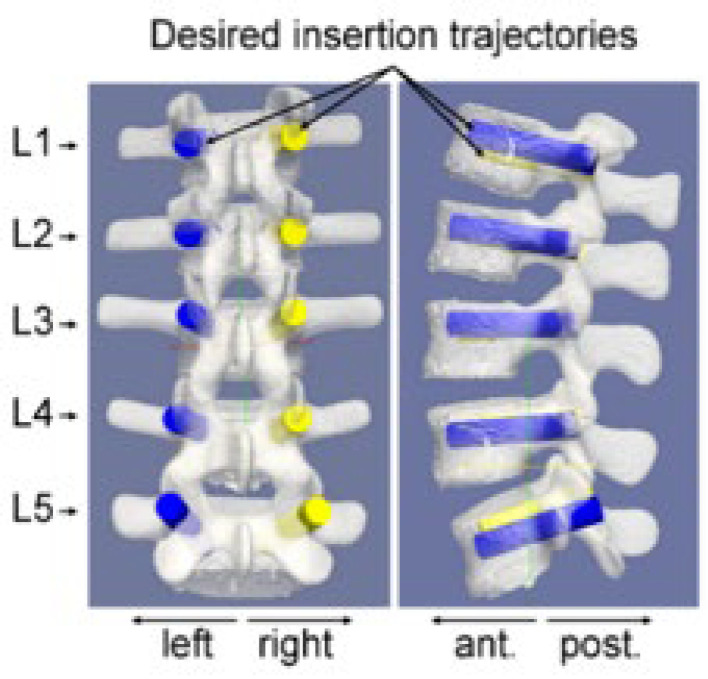
Preoperative planning of the screw insertion on the 3D CBCT model of the lumbar spine. The planning consists of ten desired insertion trajectories, including left and right pedicle screws within each of the five lumbar vertebrae (L1, L2, L3, L4 and L5). The coordinates of the entry and target points of the ten desired screw positioning are stated in the reference frame R0 of the CBCT images.

Each operator performed preoperative screw planning on three distinct lumbar spine models for each insertion method. The order of technique application was randomized for each operator to minimize bias, and a minimum one-week washout period was implemented between techniques to reduce potential carryover effects. Detailed descriptions of each insertion technique are provided in the Supplementary Materials. These software platforms provided 2D and 3D visualization of CBCT-based spine models, allowing precise positioning of screws at the center of each pedicle. For each model, operators planned ten desired screw trajectories (Figure 7), corresponding to left and right pedicle screws for each lumbar vertebra from L1 to L5. The coordinates of the entry and target points for all ten screws were recorded in the CBCT reference frame (R0).

### 2.4. Ethics Committee

The study was conducted in accordance with the Declaration of Helsinki, and approved by the Institutional Review Board of Catholic University of Louvain, Belgium (protocol code CEHF-FORM-003/REV 005 and approved on 17 April 2024).

### 2.5. Study Variables

#### 2.5.1. Collect Data

For each operator, the preoperative planning was recorded by storing the coordinates of both the entry (Pe) and target (Pt) points of the ten planned screw trajectories in the CBCT reference frame (R0). The 3D CBCT models of the lumbar spine containing the planned screws were then segmented using ITK-SNAP [30] for each model.

#### 2.5.2. Vertebral Model

**Figure 8:**
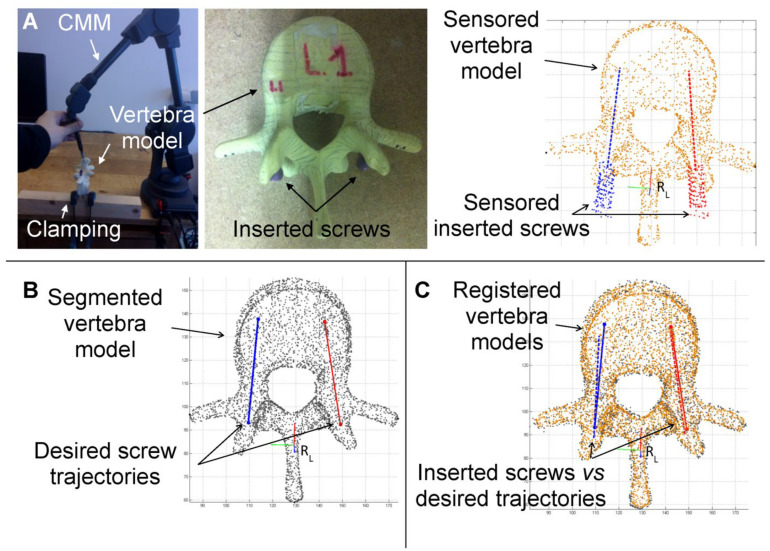
(**A**) The vertebra model with the inserted screws is digitized using a coordinate measuring machine with a spherical sensor. (**B**,**C**) The measurement coordinate set corresponding to the vertebral surface is registered to the preoperative 3D model of the same vertebra using an Iterative Closest Point algorithm using numerical computation software. The measurement coordinate set corresponding to the proximal part of the screw is fitted to a least square cylinder, and then the parameters ee, et, eα, C and dpw are calculated.

After screw insertion, each vertebral model with the inserted screws was digitized using a coordinate measuring machine (Microscribe G2X, Immersion Corporation, San José, CA, USA) with a 2 mm spherical sensor, achieving a precision of 0.2 mm (Figure 8A).

Digitization included both the vertebral surface and the proximal part of each screw, following the guidelines for ISO-based coaxiality assessment. The 2 mm sensor was configured to ignore microscopic surface roughness, and measurement points were uniformly distributed over the 3D surfaces. The minimum measurement set for the proximal part of each screw included 20 points.

The vertebral surface coordinates were then registered to the preoperative 3D model of the same vertebra using an Iterative Closest Point (ICP) algorithm implemented in MATLAB^®^ (version R2024b, The MathWorks, Natick, MA, USA) (Figure 8B,C).

The proximal screw coordinates were fitted to a least-squares cylinder, a standard procedure for ISO parameter assessment. From this fitting, all relevant metrics were calculated, including: Entry point error (ee), Target point error (et), Orientation error (eα), Coaxiality (C), and Distance to pedicle wall (dpw).

#### 2.5.3. Assessment of Breach in Terms of Gertzbein and Heary Classification

The aim of this step was to validate dpw as a quantitative metric for automatic detection of pedicle breaches.

All screws were blindly assessed by a single operator using intraoperative CBCT images and graded according to two established qualitative classification systems: Gertzbein [21] and Heary [22] (Table 1). This assessment allowed direct comparison between dpw values and established qualitative evaluations, providing validation for the dpw metric as an objective and clinically meaningful measure of screw placement accuracy.

### 2.6. Statistical Analysis

All analyses were performed using Sigmaplot version 13.3 and R version 4.3.3. For each inserted screw, the errors in entry point (ee), target point (et), orientation angle (eα), as well as the coaxiality (C) and the distance to the pedicle wall (dpw) were calculated. To evaluate the ability of coaxiality to reflect overall insertion accuracy, Spearman correlation coefficients (ρ) were computed between C and each of the errors ee, et, and eα.

The reliability of the dpw metric in assessing pedicle screw placement was compared with qualitative intraoperative CBCT assessment using both Cohen’s Kappa coefficient [31] and Gwet’s agreement coefficient [32]. Gwet’s coefficient was included as an alternative to Kappa due to Kappa’s sensitivity to prevalence, which can complicate comparisons across studies. The strength of agreement was interpreted according to Landis and Koch, with values < 0.00 indicating poor agreement, 0.00–0.20 slight, 0.21–0.40 fair, 0.41–0.60 moderate, 0.61–0.80 substantial, and >0.81 almost perfect. In addition, the diagnostic performance of dpw as a clinically relevant metric was evaluated by calculating sensitivity, specificity, positive predictive value (PPV), and negative predictive value (NPV), using intraoperative CBCT assessment as the reference standard. To assess the potential influence of operator experience, subgroup analyses were pre-specified. Comparisons between senior and junior surgeons were conducted for all parameters (i.e., ee, et, eα, C, dpw) using non-parametric Mann–Whitney U tests, chosen due to potential non-normal distribution of the metrics. All reported results, including Kappa, Gwet, sensitivity, specificity, PPV, and NPV, were presented with 95% confidence intervals to provide a comprehensive evaluation of the accuracy, reliability, and reproducibility of the proposed quantitative metrics across operators of varying experience levels.

The experiment followed a balanced factorial design with repeated measurements. Two experimental factors were considered: insertion technique (three levels: freehand, CBCT-assisted, and navigation-assisted) and operator experience (two levels: senior and resident). Four operators were included in each experience group. Each operator performed ten screw insertions per technique. The total number of screw placements therefore followed the factorial structure N = a × b × n × r, where a represents the number of techniques (*n* = 3), b the experience levels (*n* = 2), n the number of operators per group (*n* = 4), and r the repetitions per condition (*n* = 10), yielding a total of 240 screw insertions.

Comparisons between surgical guidance techniques were primarily descriptive, and the study was not powered for definitive between-technique superiority testing. These aspects should be considered when interpreting the results.

## 3. Results

Of the 240 inserted screws (30 for each of the eight operators), 236 were available for the evaluation of the insertion accuracy with conventional, CBCT-assisted and navigation-assisted techniques. Four screws were excluded because of breakage of the synthetic vertebra model during the insertion or of erroneous (backup errors) data during the preoperative planning process making it impossible to compare the positioning of the inserted screw with the desired insertion trajectory. All techniques (conventional, CBCT-assisted, and navigated) were adequately represented, although distribution was not perfectly equal due to anatomical and technical constraints.

### 3.1. Computed Insertion Errors, Coaxiality and d_pw_

The errors in entry point, target point, and orientation angle represent the trueness of the screw placement relative to the planned trajectory. The errors in entry point averaged 3.4 mm (95% CI, 3.0 to 3.8 mm) for the conventional insertion technique, 2.5 mm (95% CI, 2.1 to 2.9 mm) for the CBCT-assisted insertion technique and 2.4 mm (95% CI, 2.1 to 2.7 mm) for the navigation-assisted insertion technique.

The errors in target point averaged 5.1 mm (95% CI, 4.4 to 5.8 mm) for the conventional insertion technique, 4.2 mm (95% CI, 3.7 to 4.7 mm) for the CBCT-assisted insertion technique and 4.1 mm (95% CI, 3.7 to 4.5 mm) for the navigation-assisted insertion technique.

The errors in orientation angle averaged 7.9° (95% CI, 7.0° to 8.9°) for the conventional insertion technique, 5.2° (95% CI, 4.5° to 5.9°) for the CBCT-assisted insertion technique and 5.9° (95% CI, 5.2° to 6.0°) for the navigation-assisted insertion technique.

The coaxiality metric, representing the overall deviation in position and orientation of the screw (combining aspects of trueness and precision), averaged 5.8 mm (95% CI, 5.2 to 6.4 mm) for the conventional insertion technique, 4.7 mm (95% CI, 4.2 to 5.2 mm) for the CBCT-assisted insertion technique and 4.4 mm (95% CI, 4.0 to 4.8 mm) for the navigation-assisted insertion technique.

The distances to the pedicle wall averaged 4.0 mm (95% CI, 3.6 to 4.4 mm) for the conventional insertion technique, 4.3 mm (95% CI, 3.9 to 4.7 mm) for the CBCT-assisted insertion and 4.1 mm (95% CI, 3.7 to 4.5 mm) for the navigation-assisted insertion technique.

### 3.2. Validation of Coaxiality Parameter

When computing the Spearman correlations ρ, a dependence appeared between the coaxiality metric and the errors in entry point (r = 0.41, *p* < 0.001), the errors in target point (r = 0.85, *p* < 0.001) and the errors in orientation angle (r = 0.48, *p* < 0.001). The coaxiality of the inserted screw contained all information necessary to determine the level of positioning accuracy in terms of entry and target points and orientation angle.

Spearman correlation analyses confirmed that the coaxiality metric, representing the overall deviation in position and orientation of the screw, strongly reflected overall placement accuracy, showing correlations with trueness in entry point errors (ρ = 0.41, *p* < 0.001), target point errors (ρ = 0.85, *p* < 0.001), and orientation angle errors (ρ = 0.48, *p* < 0.001). These results indicate that the coaxiality metric effectively encapsulates the key aspects of pedicle screw positioning across operators of different experience levels.

### 3.3. Assessment of Screw Placement and Pedicle Breach

Of the 236 inserted screws, available for the screw placement assessment with Gertzbein [21] and Heary [22] classifications, 23 (9.8%) were considered pedicle breaches using the intraoperative CBCT images, including 9 breaches when using conventional insertion technique, 7 breaches when using CBCT-assisted technique and 7 breaches when using navigation-assisted technique.

Of the 236 inserted screws, 33 (14.0%) were considered pedicle breaches using the quantitative parameter distance to the pedicle wall (d_pw_), including 13 breaches when using conventional insertion technique, 9 breaches when using CBCT-assisted technique and 11 breaches when using navigation-assisted technique.

Using the Gertzbein classification [21], 14 of the 236 screws (5.9%) were graded differently using the CBCT images and the parameter d_pw_ (Table 2). One screw was graded 0 with no breach with the parameter dpw, while the CBCT technique revealed a lateral breach (grade 4). Six screws were graded 1 with a medial breach < 2mm with the parameter dpw, while the CBCT technique revealed no breach for four of them (grade 0) and a medial breach of 2–4 mm (graded 2) for two of them. Finally, two screws were graded as lateral breach (grade 4) with the parameter dpw, while the CBCT technique did not reveal breach (grade 0). Five screws were graded as superior pedicle breaches with the parameter dpw with no corresponding grade in the Gertzbein [21].

Using the Heary classification [22], 12 of the 236 screws (5.1%) were graded differently using the CBCT images and the parameter dpw (Table 3). One screw was considered grade I with no breach with the parameter dpw, while the CBCT technique revealed an in-out-in screw (grade II). Two screws were considered in-out-in screws (grade II) with the parameter dpw, while the CBCT technique revealed no breach (grade I). Finally, four screws were considered grade IV with medial breach with the parameter dpw, while the CBCT technique revealed no breach (grade I). Five screws were graded as superior pedicle breaches with the parameter dpw with no corresponding grade in Heary classifications.

Using the Gertzbein [21] and Heary [22] classifications, agreement on screw placement between the CBCT images and the parameter dpw was almost perfect with a Kappa coefficient of 0.86 (95% CI, 0.76 to 0.95) and a Gwet coefficient of 0.96 (95% CI, 0.93 to 0.99). The sensitivity of the parameter d_pw_ was 0.96 (95% CI, 0.79 to 0.99), the specificity 0.97 (95% CI, 0.94 to 0.99), the PPV 0.79 (95% IC, 0.64 to 0.94) and the NPV 0.99 (95% CI, 0.98 to 1.00).

### 3.4. Comparison of Pedicle Screw Insertion Between Experienced Surgeons (Senior) and Residents (Junior)

**Figure 9:**
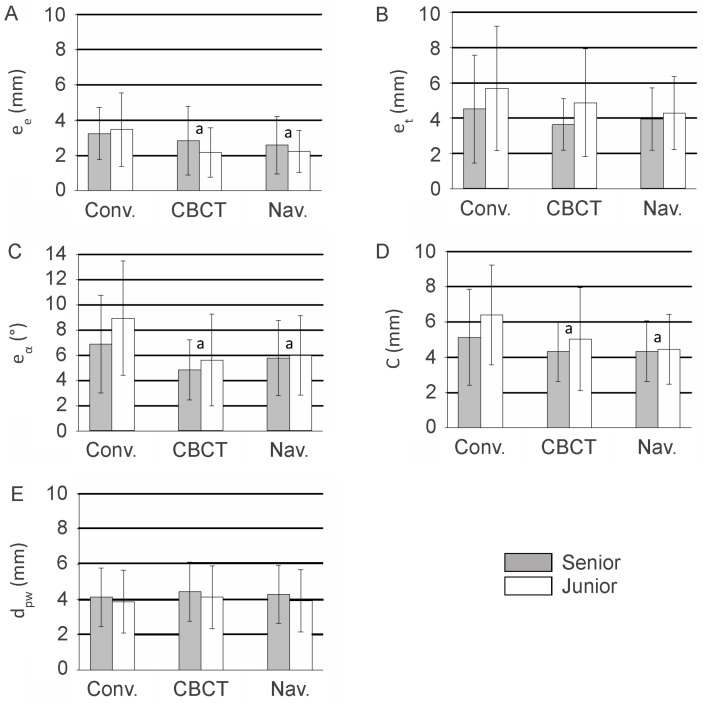
(**A**) Comparison of the errors in entry point (ee), (**B**) errors in target point (e_t_), (**C**) errors in orientation angle (e_α_), (**D**) coaxiality accuracy (named “C”) and (**E**) the distances from the axes of the inserted screws to the pedicle wall (d_pw_) achieved by the senior and the junior surgeons among the three insertion techniques (conventional, CBCT-assisted and navigation-assisted). Mean values are shown with the lower and upper limits of the 95% confidence interval. a: *p* < 0.05 compared with conventional insertion technique.

Subgroup analyses were performed to evaluate the effect of operator experience (see Figure 9). No significant difference was observed between senior and junior surgeons with respect to trueness in entry point (*p* = 0.201) or achieved distances to the pedicle wall (d_pw_) (*p* = 0.163). Small but statistically significant differences were noted for orientation angle errors (*p* = 0.025) and the coaxiality metric (*p* = 0.023), while target point errors differed significantly between the two groups (*p* = 0.006).

## 4. Discussion

This experimental study was designed according to the recent concept of quantitative orthopedic surgery [23,33,34,35,36,37,38] and represents the first study to assess pedicle screw insertion accuracy using the ISO 1101 coaxiality parameter. The main finding of this study was that the proposed dual-metric framework demonstrated strong agreement with established CBCT-based classifications while providing reproducible quantitative assessment.

This ISO-based methodology offered three main advantages. First, it consolidated all information regarding the position and orientation of the inserted screw into a single parameter, coaxiality, as confirmed by the strong Spearman correlations observed between coaxiality and errors in entry point (ρ = 0.41), target point (ρ = 0.85), and orientation angle (ρ = 0.48). This demonstrates that coaxiality effectively encapsulates the key aspects of pedicle screw positioning and enables reproducible comparisons across different levels of surgical experience.

When detailed analysis was required, the method also allowed separate assessment of insertion errors in entry point, target point, and orientation angle relative to the desired trajectory. Second, the coaxiality parameter could be applied to pedicle screw insertions across any vertebra, provided that a reference frame for measurement was defined. Third, it offered a standardized metric for comparing the performance of various assistive technologies.

The results regarding the mean and variability of the distance from the screw axis to the pedicle wall indicated that screws were consistently inserted within the pedicles. The comparison between dpw-based assessment and CBCT-based Gertzbein grading is detailed in Table 2, where 202 screws were concordantly graded as fully contained (grade 0), and overall disagreement concerned only a limited number of cases. Similarly, comparison with the Heary classification (Table 3) showed that 12 of 236 screws (5.1%) were graded differently.

Agreement analysis demonstrated almost perfect concordance between dpw and CBCT-based qualitative assessment, with a Kappa coefficient of 0.86 and a Gwet coefficient of 0.96. Diagnostic performance metrics further supported the validity of dpw: sensitivity was 0.96, specificity 0.97, PPV 0.79, and NPV 0.99. These values confirm that dpw reliably detected breaches while minimizing false negatives. Notably, the dpw parameter identified a total of 10 additional pedicle breaches that were not detected by qualitative CBCT assessment, including five superior pedicle breaches (Table 3). This highlights that traditional qualitative grading systems may underreport borderline cortical breaches, leading to false negatives.

The dpw metric provides a continuous, millimetric measure of screw proximity to the pedicle wall, enabling more sensitive detection of clinically relevant deviations and potentially facilitating immediate intraoperative correction. Future studies implementing this quantitative approach on clinical datasets are warranted to further validate these findings.

Subgroup analyses by operator experience (senior vs. junior surgeons; see Figure 8) indicated that entry point errors (*p* = 0.201) and dpw (*p* = 0.163) did not significantly differ between groups, whereas orientation angle errors (*p* = 0.025), target point errors (*p* = 0.006), and coaxiality accuracy (*p* = 0.023) showed small but statistically significant differences. These results suggest that while surgical experience may slightly influence certain aspects of screw trajectory precision, the dual-metric framework is robust across operators of varying expertise.

Quantitative assessment in spine surgery was not a new concept. Prior studies used manual or imaging-based measurements to evaluate deviations between planned and actual screw positions. Kamimura et al. [32] first described angular deviations in vivo, Oertel et al. [39] measured mean angular deviations using the O-arm in axial views only, and Mathew et al. [40] assessed angular deviations in axial and sagittal planes relative to a preoperative plan. Guha et al. [41] compared qualitative Heary classification assessment with quantitative measurements and found poor correlation between observer-based qualitative grading and absolute insertion errors.

Byeong-Jin et al. [26] also proposed a quantitative evaluation combining distance-based measurements with the Gertzbein classification [21]. However, in their study, quantitative metrics were not directly correlated with classification grades beyond reporting proportions of misplaced screws, and segmentation accuracy was not specifically validated. In addition, their method required systematic measurement of multiple error components, potentially limiting routine applicability. In contrast, the present study validated both coaxiality and pedicle wall distance against established qualitative assessments (Table 2 and Table 3), demonstrating that these ISO-based parameters provided sufficient, reproducible, and clinically relevant information for assessing pedicle screw insertion accuracy and breach detection.

This study has several limitations. Pedicle screw insertion was performed on synthetic lumbar spine models made of homogeneous polyurethane foam lacking trabecular architecture and cortical variability. While this ensured experimental standardization and high measurement reproducibility, it does not replicate the heterogeneous mechanical properties of human vertebrae. The absence of cortical thickness variation and trabecular anisotropy may have altered drilling resistance and tactile feedback, potentially influencing fine trajectory adjustments and thereby affecting pedicle wall distance (dpw) and coaxiality measurements.

Although operators worked under realistic visual and access constraints, the procedures were conducted in a controlled, non-aseptic environment without bleeding, soft tissue interference, anesthesia-related physiological motion, or patient movement. Furthermore, no metallic instrumentation was present, eliminating imaging artifacts that may degrade CBCT-based assessment in clinical practice. These optimized experimental conditions likely enhanced geometric measurement precision and may therefore overestimate the robustness and reproducibility of the proposed metrics under real surgical circumstances.

Only lumbar vertebrae were evaluated. However, pedicle morphology varies substantially across spinal regions. Thoracic pedicles are narrower and demonstrate greater angular variability, while cervical pedicles are smaller and anatomically more constrained. In deformity cases such as scoliosis, vertebral rotation, pedicle asymmetry, and dysplastic morphology further increase trajectory complexity. Because dpw is inherently dependent on pedicle diameter and angular tolerance, smaller pedicles provide a reduced geometric safety margin, meaning that minimal angular deviations may lead to proportionally greater reductions in cortical distance. Consequently, the robustness of dpw and coaxiality observed in standardized lumbar models may not fully extrapolate to thoracic, cervical, or complex deformity scenarios where anatomical tolerances are significantly reduced.

Future biomechanical and imaging validation studies across different spinal levels, including thoracic, cervical, deformity, and osteoporotic conditions, are necessary to determine whether the sensitivity and threshold values of dpw and coaxiality require level-specific adaptation and to confirm the generalizability of this dual-metric framework in real-world surgical settings.

Despite these limitations, the proposed dual-metric framework provides clinically meaningful advantages. The pedicle wall distance (dpw) offers immediate, intuitive, and clinically interpretable feedback that may assist intraoperative decision-making and facilitate early detection of cortical breaches. Coaxiality, in contrast, provides a standardized and reproducible geometric parameter suitable for objective benchmarking and comparison across surgical guidance technologies.

Beyond the standard lumbar models used in this study, the clinical value of precise and reproducible quantitative assessment is especially relevant in challenging surgical scenarios, such as osteoporotic bone or deformed vertebrae. In these cases, malpositioning is not the only concern; mechanical stability can be compromised, and augmented techniques such as fenestrated pedicle screws with polymethylmethacrylate cement are often employed to enhance fixation. Recent clinical data support the reliability of such augmentation methods in fragile bone, demonstrating effective screw stabilization and low complication rates [42]. In this context, standardized quantitative frameworks like the coaxiality and dpw metrics provide a robust means to objectively evaluate the precision of screw placement and the potential benefit of augmentation, helping surgeons anticipate risks, optimize intraoperative planning, and improve outcomes in compromised bone. The clinical relevance of precise and reproducible quantitative assessment becomes particularly important in challenging scenarios such as osteoporotic bone or spinal deformity. In these cases, malpositioning is not the only concern; mechanical stability may also be compromised. Augmentation techniques, including fenestrated pedicle screws combined with polymethylmethacrylate cement, are frequently employed to enhance fixation in fragile bone. Within this context, standardized quantitative metrics such as dpw and coaxiality provide a robust framework for objectively evaluating screw placement precision and potentially assessing the added value of augmentation strategies. By enabling structured and reproducible measurement, this framework may help surgeons anticipate biomechanical risk, refine intraoperative planning, and ultimately improve patient outcomes in anatomically and mechanically compromised conditions.

All CBCT-based measurements were performed by a single experienced evaluator. Although the proposed metrics rely on predefined geometric criteria and quantitative computation rather than subjective categorical grading, operator-dependent steps such as image segmentation and axis definition may introduce measurement variability. Inter- and intra-observer reliability were not formally assessed in this study, as methodological validation of reproducibility was not the primary objective. Future investigations should incorporate independent blinded evaluators and formal reliability testing to further strengthen validation of this quantitative framework.

## 5. Conclusions

In summary, the proposed dual-metric framework combining coaxiality and dpw provided a reproducible, quantitative, and clinically relevant assessment of pedicle screw placement accuracy. The framework demonstrated strong agreement with established CBCT-based qualitative classifications (Gertzbein–Robbins and Heary) and reliably detected deviations, including breaches not captured by traditional grading systems.

Subgroup analyses indicated that while certain trajectory parameters (i.e., target point, orientation angle, and coaxiality) were slightly influenced by surgical experience, entry point accuracy and dpw were consistent across senior and junior surgeons, highlighting the robustness of this framework for operators of varying expertise.

This dual-metric approach offers practical utility for intraoperative feedback, surgical education, and benchmarking across guidance technologies. Coaxiality encapsulates geometric accuracy for research comparisons, while dpw provides immediate, clinically actionable information to support safe screw insertion.

Finally, while the ISO-based computational method using mechanical digitization of vertebral surfaces demonstrated high precision, further studies are required to implement and validate coaxiality and dpw computation using intraoperative CBCT with surface segmentation and metallic artifact reduction, to fully extend this quantitative framework to real surgical settings and more complex vertebral anatomies.

## Figures and Tables

**Table 1:** Gertzbein and Heary classifications.

Gertzbein (G)			Heary (H)
	**G Grade**	**H Grade**	
screw fully contained within the pedicle	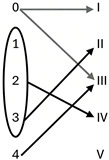		screw fully entirely within the pedicle
medial breach < 2 mm			screw violates pedicle but screw tip entirely contained within the vertebral body
medial breach 2–4 mm			screw tip penetrates anterior or lateral vertebral body
medial breach > 4 mm			breach medial or inferior pedicle
lateral breach			screw violates pedicle or vertebral body and endangers spinal cord, nerve root, or great vessels and requires immediate revision

**Table 2:** Comparison between screw placement assessment using intraoperative CBCT images and using the computed distance parameter from the axis of the inserted screw to the pedicle wall according to Gertzbein classification.

CBCT Imaging Assessment								
GRADE		0	1	2	3	4	/	Total
**Assessment using the parameter of the pedicle wall distance (d_pw_)**	**0**	***202***	0	0	0	1	0	203
	**1**	*4*	**10**	2	0	0	0	16
	**2**	*0*	0	**5**	0	0	0	5
	**3**	*0*	0	0	**0**	0	0	0
	**4**	*2*	0	0	0	**5**	0	7
	**/**	*0*	0	0	0	0	**5**	5
**Total**		208	10	7	0	6	5	**236**

“/”: screws with no corresponding grade in the classifications used.

**Table 3:** Comparison between screw placement assessment using intraoperative CBCT images and using the computed distance parameter from the axis of the inserted screw to the pedicle wall according to Heary classification.

CBCT Imaging Assessment								
GRADE		I	II	III	IV	V	/	Total
**Assessment using the parameter of the pedicle wall distance (d_pw_)**	**I**	***202***	1	0	0	0	0	203
	**II**	*2*	**4**	0	0	0	0	6
	**III**	*0*	0	**1**	0	0	0	1
	**IV**	*4*	0	0	**17**	0	0	21
	**V**	*0*	0	0	0	**0**	0	0
	**/**	*0*	0	0	0	0	**5**	9
**Total**		208	5	0	17	1	5	**240**

“/”: screws with no corresponding grade in the classifications used.

## Data Availability

All data supporting the findings of this study are included in the manuscript or are available from the corresponding author upon reasonable request.

## Supplementary Materials

The following supporting information can be downloaded at: https://www.mdpi.com/article/10.3390/jcm15062328/s1, Description of pedicle screw insertion techniques.
