# Supplementary material for: Combining Engineering Precision with Clinical Relevance: A Novel Dual Framework for Assessing Pedicle Screw Accuracy in Spine Surgery
Source: J Clin Med. 2026 Mar 18;15(6):2328. doi: 10.3390/jcm15062328 (PMC13026749; doi:10.3390/jcm15062328)
Supplement: Supplementary file 1 [file jcm-15-02328-s001.zip › jcm-4179932-supplementary.pdf]

Supplementary material: Description of pedicle screw insertion techniques.

Each operator performed preoperative screw planning on three distinct lumbar spine models assigned exclusively to that operator, using syngo DynaCT software for conventional and CBCT-assisted pedicle screw insertion and using iPlan Spine 3.0 (Brainlab AG, Munich, Germany; technique 3) for Navigation-assisted pedicle screw insertion. In practice, the planning of each operator was recorded by storing the coordinates of both the entry and target points of the ten desired screw positioning stated in the reference frame of the CBCT images.

Conventional pedicle screw insertion (technique 1)

The eight operators individually performed ten screw insertion, one within each of the left and right sides of each of the five lumbar vertebrae. Operators were instructed to respect the desired insertion trajectories previously defined as accurately as possible so as to avoid pedicle breach, however, in order to simulate realistic conditions, operators were not allowed to visualize the preoperative 3D planning during the insertions. During the insertions, each operator was free to use a conventional 2D-fluoroscopic system (BV Pulsera, Philips, Amsterdam, the Netherlands) to acquire intraoperative 2D-fluoroscopic images of the surgical site (Fig. S1A).

CBCT-assisted pedicle screw insertion (technique 2)

The eight operators performed the pedicle screw insertion with the aid of the CBCT system (Fig. S1B). Before the screw insertion, the operators were allowed to display the 3D CBCT model of the lumbar spine with the desired insertion trajectories of the screws. The operator registered the bone with the 3D CBCT model using semi-automatic patient-to-planning registration algorithms provided by the CBCT system. For each CBCT-assisted screw insertion, the operator is then capable to position a laser beam that consists of two perpendicular planes whose intersection materializes the entry point and the orientation of the desired insertion trajectory. The operators performed the screw insertion with visual feedback by positioning the tip and orientating the axis of the surgical tools in accordance to the laser beam.

Navigation-assisted pedicle screw insertion (technique 3)

The eight operators performed the pedicle screw insertion with the aid of a navigation system (CurveTM, Brainlab, Munich, Germany) (Fig. S1C). The 3D CBCT model of the lumbar spine with the desired insertion trajectories was loaded into the system. Before the screw insertion, each operator was allowed to fix a dynamic reference base (DRB) on the spinous processes of the vertebrae. This DRB is equipped with infrared-reflecting markers and was tracked by the

optical localizer of the navigation system. The operator registered the bone with the 3D CBCT model using semi-automatic surface-based algorithms provided by the navigation system. For each navigation-assisted screw insertion, the pedicle probe to drill the bones was also equipped with infrared-reflecting markers and tracked by the localizer. Using the tracking information, the navigation system is capable to display the real-time position of the pedicle probe relative to the lumbar spine model. The operators performed the screw insertion with real-time visual feedback displayed on a monitor screen.

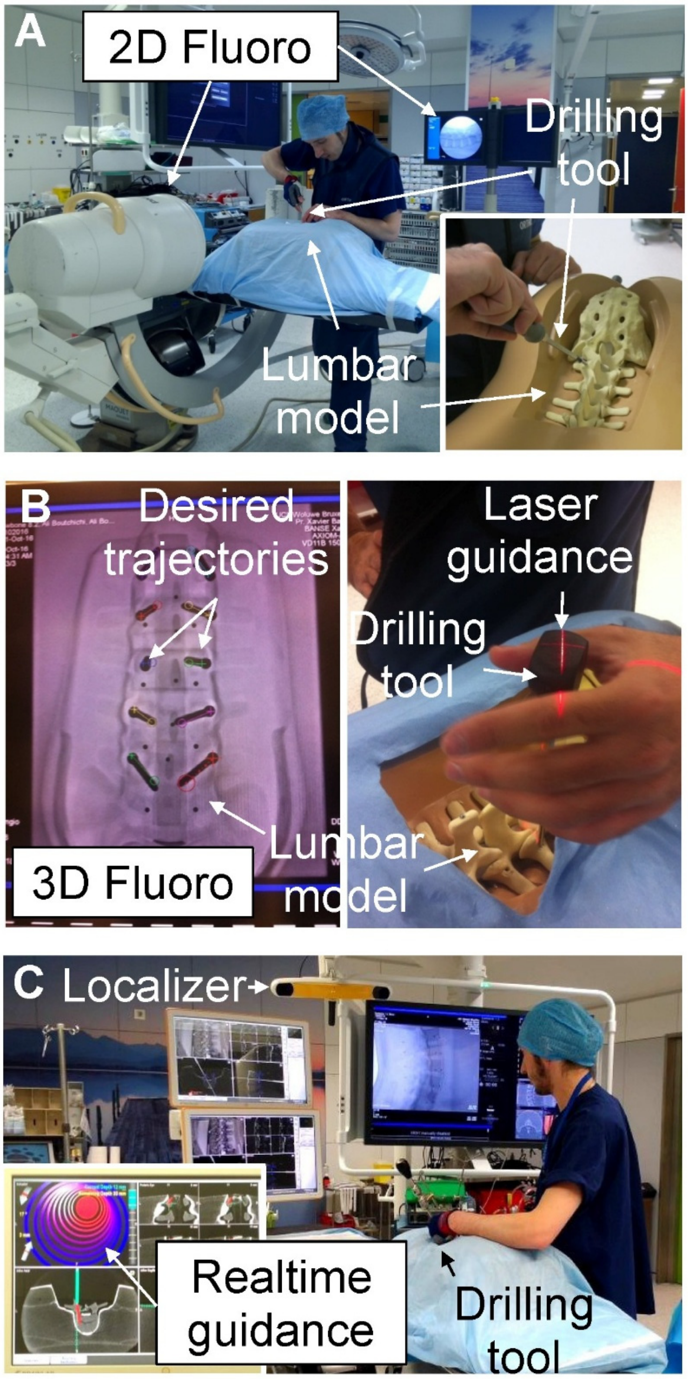

Figure S1. (A) Conventional pedicle screw insertion technique. The operator is instructed to respect the desired insertion trajectories previously defined, with free use of a conventional 2D-fluoroscopic imaging system. (B) CBCT-assisted pedicle screw insertion technique. The 3D CBCT model of the lumbar spine, with the desired insertion trajectories of the screws, is displayed on a computer screen. A laser beam that consists of two perpendicular planes materializes the entry point and the orientation of the desired insertion trajectory. The operator performs the screw insertion with visual feedback by positioning the tip and orientating the axis of the surgical tool in accordance to the laser beam. (C) Navigation-assisted pedicle screw insertion technique. The 3D CBCT model of the lumbar spine, with the desired insertion trajectories of the screws, is displayed on a computer screen. The lumbar spine model and the surgical tools are equipped with infrared-reflecting markers and tracked by the optical localizer of the navigation system. The operator performs the screw insertion with real-time visual feedback displayed on a monitor screen.
